# Homology-based identification and structural analysis of *Pangasius hypophthalmus* Annexins and Serine proteases to search molecules for wound healing applications

**DOI:** 10.1016/j.csbj.2024.10.015

**Published:** 2024-10-11

**Authors:** Maria Isabela Avila Rodríguez, Ana Julia Velez Rueda, Jesús Hernández-Pérez, Jorge Benavides, Mirna Lorena Sanchez

**Affiliations:** a Tecnologico de Monterrey, Escuela de Ingeniería y Ciencias, Ave. Eugenio Garza Sada, 2501, Monterrey, Nuevo León C.P 64849, Mexico; b Departamento de Ciencia y Tecnología, Universidad Nacional de Quilmes - CONICET, Roque Sáenz Peña 352, Bernal, Buenos Aires B1876, Argentina; c Tecnologico de Monterrey, Institute for Obesity Research, Ave. Eugenio Garza Sada 2501, Monterrey C.P 64849, Mexico; d Laboratorio de Farmacología Molecular, Departamento de Ciencia y Tecnología, Universidad Nacional de Quilmes, Roque Sáenz Peña 352, Bernal, Buenos Aires B1876, Argentina

**Keywords:** Pangasius hypophthalmus, Homology-based identification, Wound healing, Serine proteases, Annexins

## Abstract

Chronic wounds and burns are a worldwide healthcare problem that erodes patients’ well-being and healthcare systems. This silent and costly epidemic requires new, cost-efficient solutions to improve patients’ physical and economic welfare. Eschar-degrading vegetal and bacterial proteases have been utilized as a solution. However, these proteins are evolutionarily far from those present in human wound healing. Serine protease (SP) and annexin (ANX) proteins interact within the skin healing process. A homology-based identification pipeline can help in discovering selective human SP and ANX analogs in the epithelial tissue of the fast-healing species, *Pangasius hypophthalmus*. In the present work, we found 14 candidates for RT-PCR in *P. hypophthalmus* using homology inference. The genetically detected candidates were then structurally and sequentially analyzed to understand their possible relation to SPs and ANXs involved in human wound healing. A total of six TBLASTN/BLASTX candidates (four SPs and two ANXs) were detected in *P. hypophthalmus* skin. Structural analysis revealed that all SP candidates resembled human KLK4, KLK5, KLK6, and KLK8, whereas all ANX only resembled human ANXA4. Structure and sequence analysis revealed high conservation of ANX Ca^2+^ binding sites (GDXD) and SP catalytic triad (HDS) motifs. In addition, structural analysis revealed that SP substrate selectivity position 186 was the main difference between human KLK5 and *P. hypophthalmus* SPs. These findings may allow the proposal and testing of more selective formulations, broadening treatments beyond debridement.

## Graphical Abstract

**Figure:**
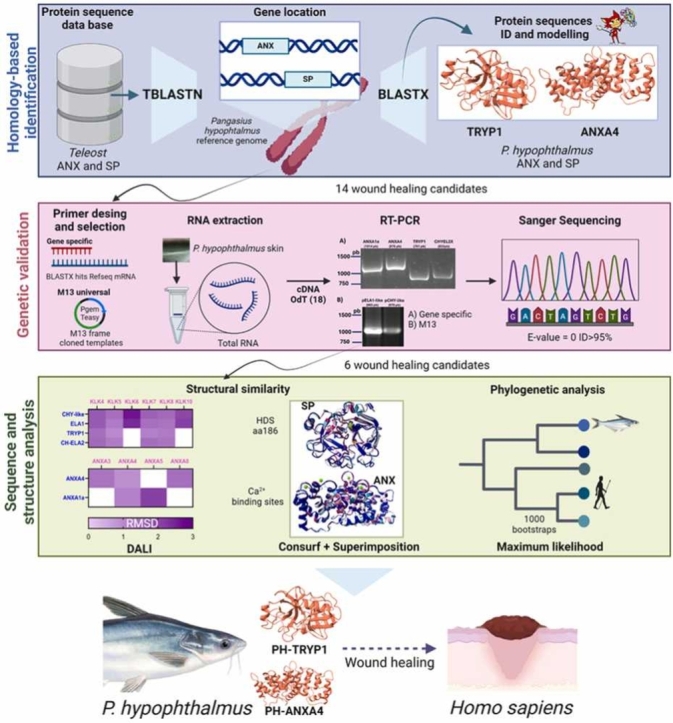
Homology-based identification and structural analysis of *P. hypophthalmus* annexins and serine proteases to search molecules for wound healing applications. This image was created with BioRender.com.

## 1 Introduction

Burns and chronic wounds are injuries that represent a burden for patients and healthcare systems worldwide. This silent epidemic encloses 8 million burn cases annually, with chronic wounds causing 2 % of global hospitalizations [1], [2]. Healing in chronic wounds is prolonged (more than 3 months), dramatically increasing patient care expenses, given 85 % of the cost related to hospital care and nursing time and out-of-pocket expenses related to home wound care, which usually is not subsidized [3], [4]. Hence, searching for new solutions that enhance the wound healing rate is necessary to improve patients’ welfare.

Debridement is one of the first steps in treating burns and chronic injuries, as it eliminates necrotic tissue (eschar) through different means, including surgical, biological, enzymatic, and autolytic [5] approaches. Often, patients require maintenance debridement as chronic to acute wound transition does not always occur. Enzymatic debridement offers a cost-effective and selective alternative, as proteases with broad extracellular matrix substrate affinity degrade eschar without requiring surgical devices [6], [7], [8]. However, despite the increasing demand for better options, only vegetal and bacterial-sourced proteases have been available for the last 30 years [9], [10], looking aside the relation with proteases involved in wound healing cascades.

Currently, biochemical screening of natural sources is the most used strategy in discovering these molecules; however, it requires expensive and labor-intensive processes [11]. Other alternatives include proteomic and genomic approaches. Nonetheless, despite allowing the confirmation of amino acid chains, proteomic analyses reveal sample-specific challenges, such as protein copy number, protein solubility, sample preparation quality, and environmental and life stage factors affecting protein expression within the biological sample, among others [12], [13], [14]. Moreover, the accelerated growth of omics data and inadequate analysis pipelines may lead to false-positive assumptions [15].

Specific molecular mechanisms in healthy and altered wound healing pathways are well elucidated; therefore, homology-based techniques offer an adequate solution for searching novel alternatives. This information describes which type of proteases and other molecules participate in these cascades [16]. This allows the selection of key wound healing protein sequences as queries to search for new wound healing candidates in validated genomic, transcriptomic, and protein sequence databases, instead of the usual extensive biochemical assessment.

Different proteins have been found to act in various steps that aid wound closure. For example, annexins (ANXs) have been reported to allow plasma membrane closure, antithrombosis, and cell migration and to exhibit anti-inflammatory capacities [17], [18], [19]. At the same time, serine proteases (SPs) have been directly related as participants and activators during desquamation and the four stages of wound healing (hemostasis, inflammation, migration/proliferation, and tissue remodeling) [20], [21]. Both ANXs and SPs have been identified in the teleost integumentary system [22], [23], [24].

Teleost fish, such as *Pangasius hypophthalmus*, has been suggested as a promising organism for research, as this species has been reported to achieve full scarless recovery from skin excision (5 × 4 cm) in 4 weeks without therapeutic intervention, besides having a comparable wound healing process to nonaquatic animals [25]. This is in comparison to humans, where skin wound healing occurs from 4 to 6 weeks, with a scar maturation period of a year to several years depending on the wound type [26], [27]. In addition, there is a similarity between human and fish skin structures and functions, including layers, gland-produced secretions with pathogen defense-related molecules, collagen type I, V, and VI expression, and shared inflammation signaling pathways [28], [29]. Because of these similarities, searching for human analogs of SPs and ANXs in *P. hypophthalmus* through homology-based identification could help in finding wound healing candidates with a more specific relation to the human skin context. Hence, this study aims to locate the presence of ANX and SP families in *P. hypophthalmus* genome through homology-based identification and understand their similarities with their human skin homologues through structural and phylogenetic analysis.

## 2 Materials and methods

### 2.1 TBLASTN protein sequence set formation and curation

The protein to translated nucleotide BLAST (TBLASTN) 2.11.0 + tool was used to locate ANX and SP in *P. hypophthalmus* genome [30]. For this, well-annotated extracellular ANX and SP sequence sets from teleosts were generated to be used as a TBLASTN query. These protein family sequences were retrieved from the UniProtKB search engine using keywords such as serine proteases, annexins, and kallikreins [31]. A taxonomy filter was used to narrow the results, with the inputs Teleostei (9TELE) [32443] and Siluriformes (catfishes) [7995] used to include only bony fish-related proteins. From each search, proteins that fell under the reviewed annotation category and had records of extracellular expression in the skin were selected. Proteins that were fragments, inhibitors, or were not properly annotated were discarded. Later, each sequence was evaluated through the PROSITE tool (https://prosite.expasy.org/) [32] to identify the specific domains per type of protein before further blast analyses.

### 2.2 Protein locus and sequence identification

The UniProtKB ANX and SP sequence sets were used as a query against the *P. hypophthalmus* reference genome (GCF_027358585.1) from the bio project PRJNA914796 for TBLASTN analysis. The whole amino acid sequence was used for SPs, whereas only the first domain of each sequence was used for ANXs, as the sequence contains four repeats of the same domain. A default search setup was held with an expanded maximum target of 5000. The resulting hits were curated by selecting those with an E-value < 1 × 10^−3^ [33] and an identity percentage of 50 % or higher. The NCBI sequence viewer tool was used to obtain the coding sequence (CDS) length and amino acid sequence length of TBLASTN hits [34].

The curated hits were then used as a query in BLASTX 2.12.0 + [30] using a default configuration. The results obtained from *P. hypophthalmus* with a 100 % identity and an E-value lower than 1 × 10^−3^ were selected as candidates for further analysis and genetic validation. Unrelated *P. hypophthalmus* hits and nonsignificant hits were discarded.

### 2.3 Primer design

Primers were designed through the Primer3Plus [35] using the CDSs of RefSeq mRNA from BLASTX hits as templates. Primer quality criteria were held as reported by Apte et al. [36]. Primers with 3′ mismatches greater than one nucleotide were also disregarded from further analysis [37]. Primer specificity analysis was held through the Primer Blast tool enclosing the search to *P. hypophthalmus* (taxid:310915) in “Primer Pair Specificity Checking Parameters” to verify if the designed primers amplified the desired targets [38]. Table 1 presents the primers designed from TBLASTX hits for genomic detection through RT-PCR.

**Table 1:** List of primers used to detect *P. hypophthalmus* SPs and ANXs found through TBLASTX analysis.

**Sequence**	**Primer name**	**Annealing temperature (ºC)**
**Serine proteases**		
5′ - GATCAGCACCGACTCCAAAT- 3′	TRYP1Fwd	60 °C
5′ - TTAATTGCGGCTCATGGTGT - 3′	TRYP1Rev	
5′ - ACATGAAGCTCTTGGTCTTGG - 3′	CHELX2Fwd	62 °C
5′ - GCTGATGAAGCACTCCAGTTT - 3′	CHELX2Rev	
5′ - AGTCATGCTGAGATTCCTTTTG - 3′	ELA1Fwd	60 °C
5′ - CATCCAGTTTATCCAACAATGC - 3′	ELA1Rev	
5′ - ATGGCTCTCCTGTGGATTCTCT - 3′	CHYA46Fwd	62 °C
5′ - TTAGTTGGAGGCAATGGTCTGAT - 3′	CHYA46Rev	
**Annexins**		
5′ - ATGGCAGCGCTTGGAAAC - 3′	ANXA4Fwd	62 °C
5′ - GACTCGTGGCTTAGATTTCTGCT - 3′	ANXA4Rev	
5′ - ATGGCTTTCATCAGCGAGTT- 3′	ANXA1Fwd	
5′ - TTACTGATCACTTCCACACAACG - 3′	ANXA1Rev	
**M13 universal primers**		
5′ - GTAAAACGACGGCCAGTG - 3′	M13 −20	56 ºC
5′ - AGCGGATAACAATTTCACAC - 3′	M13 −48	

### 2.4 Sample collection and pretreatment

Skin samples were collected under sterile conditions from seven *P. hypophthalmus* juvenile specimens (6 months to 1 year) within a maximum of 24 h after death from natural causes (bioethics permit no. 2022–10 granted by the *Comité Institucional para el Cuidado y Uso de Animales de Laboratorio* from Tecnologico de Monterrey). Approximately 120 mg ± 34 of skin with no muscle was then stored in five volumes of RNAlater reagent (Thermo Fisher Scientific, Waltham, MA) at 4 ºC for 24 h. The supernatant was discarded, and skin tissue was stored at −80 ºC until further use.

### 2.5 Total RNA extraction and cDNA synthesis

Total RNA was extracted from < 30 mg of tissue using RNeasy Mini Kit (QIAGEN, Hilden, Germany) according to manufacturer instructions. Tissue disruption was performed using liquid nitrogen in a mortar under sterile conditions. RNA was eluted in 30 µL of RNase-free water. Purity and quantity were determined through NanoDrop 1000 and Qubit™ 4 Fluorometer using RNA HS Qubit kit (Thermo Fisher Scientific, Waltham, MA). The cDNA synthesis from total RNA was then obtained using a RevertAid kit (Thermo Fisher Scientific, Waltham, MA). For this, 20 µL reactions were performed using 5 µM Oligo(dT)18 base primers and 400 ng of total RNA. The thermocycling program was 42 ºC for 60 min and 70 ºC for 5 min. After the first-strand synthesis, samples were stored at −80 ºC until further use.

### 2.6 Polymerase chain reaction

PCR amplifications were performed using 1 µL of cDNA as a template and 1 µM gene-specific primers (Table 1) with Platinum Taq polymerase (Thermo Fisher Scientific, Waltham, MA) in 10 µL reactions with 1% DMSO [39]. Thermocycling conditions were set as follows: 3 min at 95 ºC, 30 cycles with a 95º C denaturation for 30 s, specific annealing temperature according to each primer set (Table 1) for 30 s, a 68 ºC extension for Platinum Taq for 1:45 min, and a final extension of 5 min at the respective temperature. All high-fidelity PCR products were run in a 1.2% agarose gel, purified through Wizard SV Gel and PCR Clean-up kit (Promega), and stored at −20 ºC until further use. NanoDrop 1000 was used to assess the purity and concentration of purified amplicons.

ELA1 and CHY46 primer pairs (Table 1) were used with the high-fidelity polymerase PrimeSTAR (Takara, Takara Biotech Co. Ltd., Dalian). The fragments obtained were ligated overnight at 4 °C to a pGEM-T Easy (Promega) backbone. A 1:1 product-to-plasmid ratio was used in 10 µL reactions. Ligations were propagated [40], [41] and purified (pELA1 and pCHY-like) through the Wizard Plus SV kit (Promega) using a 30 µL nuclease-free water elution. Samples were stored at −20 ºC until further use. PCR product cloning confirmation was done *via* PCR using GoTaq Master Mix, with either 20 ng of pELA1 or pCHY46 as templates, and M13 forward (−20) and reverse (−48) universal primers (Table 1). All PCR size confirmations were run through 1.2% agarose gel at 90 V and stained with GelGreen (Biotium) following manufacturer instructions.

### 2.7 Sanger sequencing

Amplicon sequencing of BLASTX candidates was performed with BigDye® Direct Cycle Sequencing Kit (Life Technologies Corporation, Carlsbad, CA) according to manufacturer instructions. Reactions were then purified with BigDye™ XTerminator according to manufacturer instructions. Samples were processed using a SeqStudio Genetic Analyzer (Thermo Fisher Scientific, Waltham, MA), and results were validated through BLASTN using default settings and MegaBLAST algorithm [30].

### 2.8 Structural modeling and analysis

The structural analysis was performed modeling BLASTX hit sequences using ColabFold v1.5.5: AlphaFold2 with the MMseqs2 algorithm [42]. The models with predicted local distance difference test (pLDDT) scores > 80 and predicted template modeling (ptm) scores > 0.5 [43] were used as queries for structural similarity analysis with the DALI server [44], [45], [46]. Protein data bank (PDB) search algorithm and a cut range of PDB50 were used with standard settings [44]. The *Homo sapiens* hits chosen for further phylogenetic and structural superimposition analysis through UCSF-Chimera [47] were those related to wound healing processes with a Monte Carlo optimized similarity score (Z-score) > 4, root mean square deviation (RMSD) of Cα–Cα positions between structures < 2, and an ID% > 30. ConSurf software was used to locate specific structural conservation regions [48], [49]. The local RMSD for Ca^2+^ binding sites was calculated through PyMOL 3.0.3, using amino acids present in the Ca^2+^ motif loops. The isoelectric point and molecular weight of the candidates were calculated with the Expasy Compute pI/Mw tool.

### 2.9 Phylogenetic analysis

The NCBI PSI-BLAST software was used to generate the sequence set construction for phylogenetic analyses [30]. The sequence candidates were found in three iterations using the modifications of the following parameters: max target sequence of 5000, expected threshold of 1 × 10^−3^, PSI-BLAST threshold of 1 × 10^−40^, and low complexity filter masking. *Homo sapiens* ANX (ANXA5 and ANXA4) and SP (KLK5, KLK7, and KLK8) sequences obtained from DALI analysis with a reported relationship to wound healing processes [18], [50], [51] were used as queries.

The sequences were clustered through the CD-HIT tool (http://weizhong-lab.ucsd.edu/cdhit-web-server/cgi-bin/index.cgi), using default parameters and a cutoff bigger than 90 % homology for redundancy reduction [52]. Sequences with lengths lower than the queries or with low-quality descriptions were discarded from the analysis. A multiple sequence alignment (MSA) was then performed using the MAFFT algorithm [53] with the standard setting and Jalview (version 2.11.3.2) [54]. After curation, the protein sequences of confirmed RT-PCR *P. hypophthalmus* candidates, along with an outgroup sequence (30 % of identity toward human sequences to avoid spurious rooting [55]), were added to the MSA for ANXs (Figure S.7) and SPs (Figure S.8). The maximum likelihood trees were generated through IQ-TREE (1.6.12) using 1000 bootstraps with ultrafast bootstrap settings and ModelFinder [56]. The obtained results were visualized for analysis using Figtree v.1.4.4 [57].

## 3 Results and discussion

### 3.1 TBLASTN sequence set curation

A sequence set for ANXs and SPs under the previously described criteria was made using the UniProtKB search engine to identify the genomic locations of ANXs and SPs in the *P. hypophthalmus* genome (GCA_009078355.1). Only 25 sequences accomplished the curation criteria. The sequences included 11 ANXs (supplementary Table **S.1**) and 11 SPs (supplementary Table **S.2**).

### 3.2 ANX and SP sequence identification and genetic validation

The curated sequence set yielded a total of ten SP and four ANX sequences from the TBLASTN/BLASTX pipeline **(**Table 2**)**. It was observed that SPs were found in a greater number of chromosomes in the *P. hypophthalmus* genome compared with ANXs. It has been reported that SPs are more ubiquitous in vertebrates as they perform several functions, including skin homeostasis [58]. In the case of teleosts, SPs were mainly detected as part of skin complement proteases [59], [60]. Conversely, ANXs work specifically on membrane remodeling processes and signaling [61], elucidating the identification of fewer ANX homologues in the genome.

**Table 2:** Summary of ANX and SP genes and proteins found in the *P. hypophthalmus* genome using the TBLASTN/BLASTX pipeline. CDS nt, coding sequence nucleotides; AA, amino acid; Chr, chromosome. All BLASTX sequences had a 100 % ID score.

**Gene**		**Protein**			
**Chr. accession**	**CDS nt**	**Accession**	**AA length**	**Annotated protein**	**E-value**
NC_069710.1 (Chr 1)	741	XP_026769278.1	246	Trypsin 1	1.57E^−27^
NC_069711.1 (Chr 2)	840	XP_026801323.2	279	Serine protease 27-like	1.95E^−04^
NC_069717.1 (Chr 8)	804	XP_026776282.1	267	Chymotrypsin-like elastase family member 2 A isoform X1	2.76E^−13^
	801	XP_026776291.1	266	Chymotrypsin-like elastase family member 2 A isoform X2	2.85E^−13^
NC_069718.1 (Chr 9)	792	XP_026774546.1	236	Chymotrypsin A-like	5.76E^−15^
NC_069722.1 (Chr 13)	885	XP_034166078.1	294	Trypsin-like	5.05E^−24^
NC_069723.1 (Chr 14)	783	XP_026790710.1	260	Chymotrypsin-like protease CTRL−1	2.01E^−09^
NC_069729.1 (Chr 20)	807	XP_026788217.1	268	Chymotrypsin-like elastase family member 1.6 (previously elastase−1-like)	2.70E^−12^
NC_069730.1 (Chr 21)	1962	XP_053083333.1	653	Serine protease 56	4.12E^−15^
NC_069734.1 (Chr 25)	792	XP_026774546.1	263	Chymotrypsin A-like	4.65E^−11^
NC_069726.1 (Chr 17)	966	XP_026798592.1	321	Annexin A4	4.34E^−11^
NC_069733.1 (Chr 24)	1014	XP_026795943.1	337	Annexin A1a	3.41E^−11^
NC_069736.1 (Chr 27)	1047	XP_026777397.2	348	Annexin A1-like	1.23E^−08^
NC_069737.1 (Chr 28)	954	XP_053087076.1	317	Annexin A5b	9.30E^−08^

When analyzing the length of candidate CDSs, most SPs ranged between 741 and 885 bp, except SP 56 with 1962 bp (Table 2). As the CDS lengths of human kallikreins (H-KLK), SPs related to wound healing [62], ranged between 735 and 882 bp [63], further analyses continued disregarding SP 56. On the other hand, all ANX candidates had a CDS length within similar ranges when compared to *Homo sapiens* ANX1a of 1041 bp (NM_00700.3) and ANXA4 of 966 bp (NM_001153.5), keeping all ANXs for subsequent analyses.

**Fig. 1:**
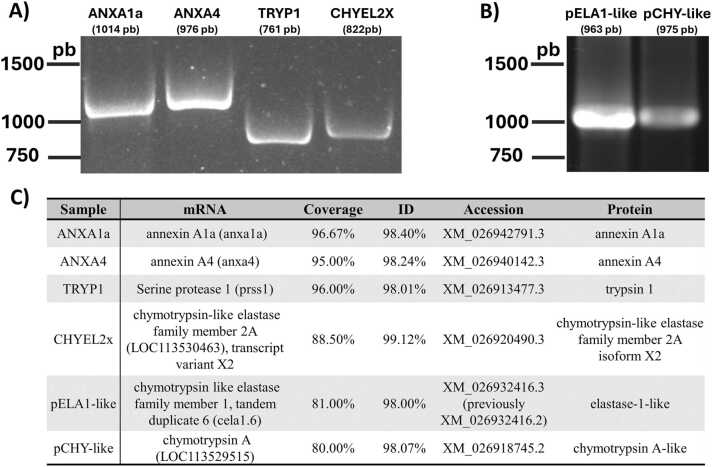
**RT-PCR detection of ANX and SP candidate from*****P. hypophthalmus*****skin.** Platinum Taq RT-PCRs from **A)** annexin A1a (ANXA1a), annexin A4 (ANXA4), trypsin 1 (TRYP1), and chymotrypsin-like elastase X2 (CHYEL2X) from cDNAs using gene-specific primers and **B)** elastase-1 (pELA1) and chymotrypsin-like (pCHY-like) amplicons using M13 primers, ran in a 1.2 % agarose gel at 90 V and revealed with GelGreen (Biotium). **C)** BLASTN average results from forward and reverse Sanger sequencing of gel-purified ANX and SP fragments.

To validate the relationship between candidates and their wound healing capability, we looked for their expression in *P. hypophthalmus* skin through mRNA RT-PCR. Of the 14 potential sequences, six sequences (four SPs and two ANXs) were detected within the epithelial tissue (Fig. 1**A**). The amplicons obtained through gene-specific primers matched the expected size of the reported CDS (Table 2).

Despite achieving gene-specific amplification as the other genes, elastase-1-like (ELA1) and chymotrypsin A-like (CHY-like) sequences did not yield enough DNA for Sanger sequencing after gel purification. Hence, the fragments obtained in the first round of PCR were re-amplified and cloned into a pGEM-T Easy (Promega) backbone. After propagation and purification, ELA1 and CHY-like sequences were amplified (Fig. 1**B**) through M13 universal primers. The amplicon size increased to 144 bp in the case of chymotrypsin-like elastase family 1.6 (ELA1) and 183 bp in the case of chymotrypsin A-like (CHY-like) due to the overhangs flanking the insert, added by cloning in the pGEM-T Easy vector.

The identity of the amplicons was confirmed through Sanger sequencing within both priming directions. After the BLASTN analysis, fragment identity matched with the TBLASN/BLASTX pipeline candidates with an exact sequence match (E-value = 0) and an identity percentage higher than 98 % (Fig. 1**C**). Coverage decrease in the case of gene-specific primers could be related to the Sanger sequencing process, where 13–40 bp is lost at the beginning as it corresponds to primer binding sites ^64^. Coverage was 100 % in the case of M13 primer amplicons as M13 priming sites included the whole target sequence. This analysis showed that the found ANXs and SPs are expressed in the skin of *P. hypothalamus* and, thus, are related to skin processes.

### 3.3 Structural and evolutionary analysis

As protein structure determines its function [65], structural analysis in functional motifs could aid in understanding the possible roles of *P. hypophthalmus* SPs (PH-SPs) and *P. hypophthalmus* ANXs (PH-ANXs) in wound healing by comparing them with *H. sapiens* SPs (H-SPs) and *H. sapiens* ANXs (H-ANXs). Genetically validated PH-SPs and PH-ANXs were then modeled using AlphaFold (**Figure S.1–S.6**) and contrasted using the DALI server to determine their structural similarities to their human counterparts in wound healing.

**Fig. 2:**
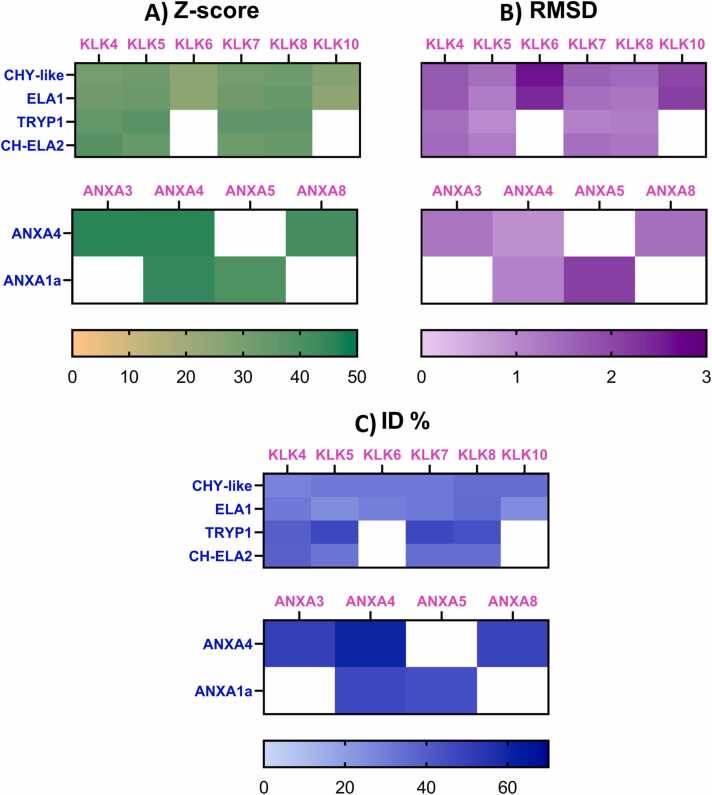
**DALI analysis score matrix of*****P. hypophthalmus*****SP and ANX candidates compared to*****H. sapiens*****through A) Z-scores, B) RMSD, and C) identity percentage (ID%) scores from PDB50.** Letters in blue and pink represent *P. hypophthalmus* proteins and *H. sapiens* DALI hits, respectively. Green, purple, and blue scale bars correspond to Z-score, RMSD, and ID% parameters, respectively.

High structural conservation was observed between *P. hypophthalmus and H. sapiens* SPs and ANXs (Fig. 2**A**). In addition, the difference between the total number of residues (NRES) and the length of aligned residues (LALI) for ANXs and SPs ranged from 2 to 8 residues **(**Table S3 **A and B)**, which means that the structural similarity comparison comprises most of the PH-ANX and PH-SP structures.

All PH-SPs showed high structural conservation with H-KLK4, H-KLK5, H-KLK7, H-KLK8, and H-KLK10, with H-KLK5 being the most conserved (Fig. 2**B**). KLKs are epidermal SPs that participate in the four overlapping steps of wound healing [62]. Specifically, H-KLK5 intervenes directly in fibrin clot remodeling through plasmin generation, provisional extracellular matrix deposition, and keratinocyte migration [62]. Meanwhile, H-KLK7 and H-KLK8 primarily intervene during cell migration [62].

Both PH-ANXs share a structural similarity with H-ANXA4 (Fig. 2**A**). H-ANXs are involved in cell wound healing through anionic membrane sealing and remodeling in a calcium-dependent manner [66]. Specifically, H-ANXA4 engages the damaged membrane by curving wound edges through self-trimerization [66]. Meanwhile, H-ANXA1 has been described as a pro-resolving agent during inflammation through reduced endothelial activation, phospholipase A2 inhibition, formyl peptide binding, efferocytosis, and leukocyte infiltration, among other mechanisms [67], [68].

The obtained Z-score and RMSD values (Figs. 2**A and** 2**B**) suggest that PH-SPs and PH-ANXs have a structural resemblance with those present in *H. sapiens* wound healing processes.

Nevertheless, when comparing the identity percentages (ID%) of PH-SPs and PH-ANXs with their human homologues, most PH-SPs exhibit ID% between 25 % and 47 %. In contrast, the ID% of PH-ANXs ranged higher between 45 % and 60 % (Fig. 2**C**). This can be explained as the protein function conservation leads to the preservation of essential structural and functional motifs along different taxa ^69^. For instance, hemoglobins from *Lumbricus terrestris* (2GLT) and *Paramphistomum epiclitum* (1H97) preserved the oxygen binding and transport capability with a 2.3 Å RMSD and a 12.1 % sequence identity [69]. Hence, a structural and sequence analysis was performed for PH-SP and PH-ANX candidates through ConSurf to understand if the structural conservation between human and PH-ANXs and PH-SPs involves the preservation of functional motifs. H-KLK5 (2PSX) and H-ANXA4 (2ZOC) crystals were used for the comparison, as these showed the highest structural conservation (Z-score and RMSD) when compared to PH-ANXs and PH-SPs, according to DALI analysis.

**Fig. 3:**
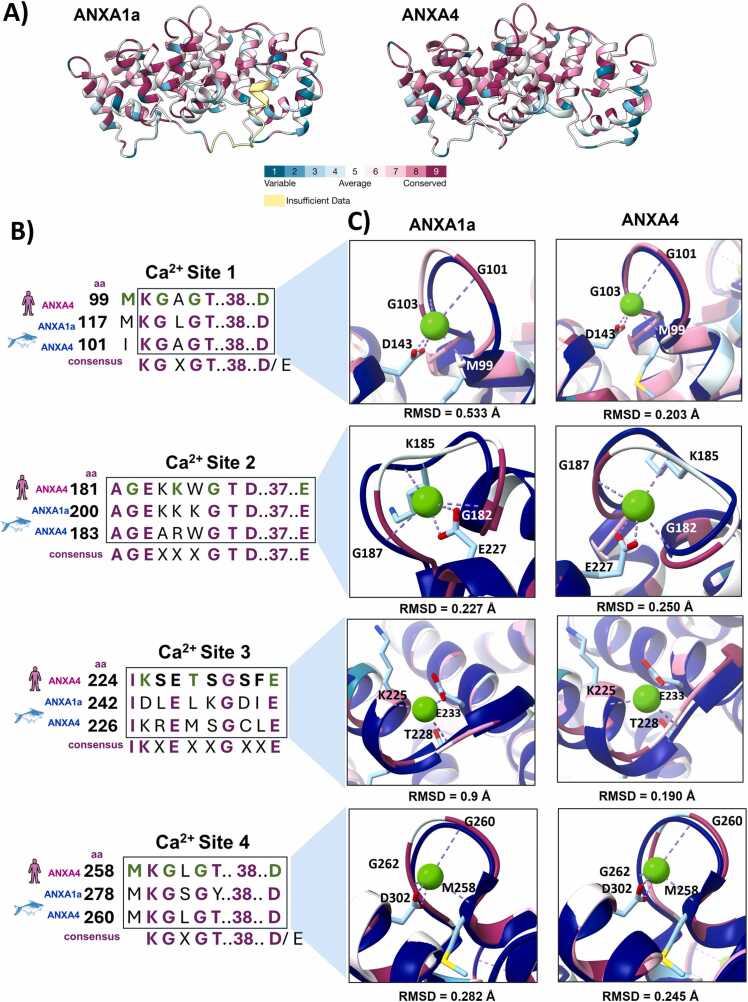
**Structural conservation of*****P. hypophthalmus*****ANX calcium sites.** A). ConSurf analysis of *P. hypophthalmus* ANXA1a and ANXA4 models. B) Motif conservation of calcium sites of *H. sapiens* ANXA4 (2ZOC) *and P. hypophthalmus* ANXA1a and ANXA4 models. Ca^2+^ coordinating residues are denoted in green. C) Superimposition of Ca^2+^ site structures for *H. sapiens* (blue navy) and *P. hypophthalmus* (ConSurf color scheme) ANX. Residue numbering and Ca^2+^ coordination sites correspond to ANXA4 (2ZOC) [64]. Local RMSD of calcium sites were calculated through PyMOL 3.0.3.

In the case of ANXs, Ca^2+^ binding sites allow the calcium-dependent binding to membrane anionic phospholipids during membrane injury and wound healing [66]. From the ConSurf analysis (Fig. 3**A**), it can be observed that PH-ANXA4 and PH-ANXA1a have a high structural and sequence conservation index at the four Ca^2+^ binding sites depicted for H-ANXA4 resolved crystal.

A comparison between candidate PH-ANXs against H-ANXA4 Ca^2+^ binding sites was then conducted through primary sequence alignment (Fig. 3**B**) and structural superimposition (Fig. 3**C**). The compared proteins contained three ANX Ca^2+^ binding sites (sites 1, 2, and 4) with a typical discontinuous α-helix–loop pair that resulted in a local RMSD between 0.190 Å and 0.250 Å for PH-ANXA4 and between 0.227 Å and 0.533 Å for PH-ANXA1a in comparison to H-ANXA4 (Fig. 3**C**). Moreover, one EF-like binding site (site 3) was detected, with a continuous α-helix–loop–α-helix structure and a local RMSD of 0.190 Å and 0.9 Å for PH-ANXA1a and PH-ANXA4, respectively. The EF-like site found for PH-ANXs has noncanonical motifs and a smaller loop than the typical 12–14 aa in EF-hands (Fig. 3**C**) [70], [71], [72]. From the primary sequence (Fig. 3**B**), it can be observed that sites 1 and 4 are canonical type II Ca^2+^ binding motifs (K**G**X**G**T**D**…37…**D/E**) [73], with well-preserved loop structure between the three proteins (Fig. 3**C**). The canonic Ca^2+^ binding motif in site 2 changes by an extension between G and GT amino acids (A**G**EXXX**G**T**D**…37…E) for all compared proteins.

These results show that the Ca^2+^ coordination of PH-ANXA4 and PH-ANXA1a may be similar to H-ANXA4 since they share high structural similarity and preserve the 2–3 carbonyl oxygen atoms from the protein backbone (M, G, and G at H-ANXA4) and a D/E carbonyl at coordination site, which allows Ca^2+^ bridging [74], [75]. Type II binding sites give selective binding to phosphatidyl serine (PS) [76]. Rosenbaum S. et al. (2011) demonstrated this selectivity, where recombinant ANXA3, ANXA4, ANXA5, ANXA8, and ANXA13, which contained the characteristic **G**x**G**T**D** motifs as PH-ANX candidates (Fig. 3**B**), bounded only to PS (100–500 pmol) and PS-containing liposomes (PC:PE:PS ratio of 7:2:1) [76].

In addition, PH-ANXs preserve aspartic acid at the **G**xGT**D** motif, which proved to be critical for Ca^2+^-bridge membrane binding ^76^. For instance, ANXA8, with a **G**xGT**K/R** substitution in PH-ANX sites 1 and 4 locations (Fig. 3**B**), lacked PS binding capability [76]. Usually, H-ANXA4 mediates cell membrane wound closure in cooperation with H-ANXA6, achieving wound contraction with H-ANXA6 and damaged cell membrane excision in a 10–15 s span [77]. Moreover, H-ANXA1 mediates inflammation by inhibiting phospholipase A2 activity, macrophage nitric oxide production, and neutrophil migration through formyl peptide receptor desensitization [77]. Hence, PH-ANXs may be tested for these activities to understand their capability to interact during cell membrane wound healing.

**Fig. 4:**
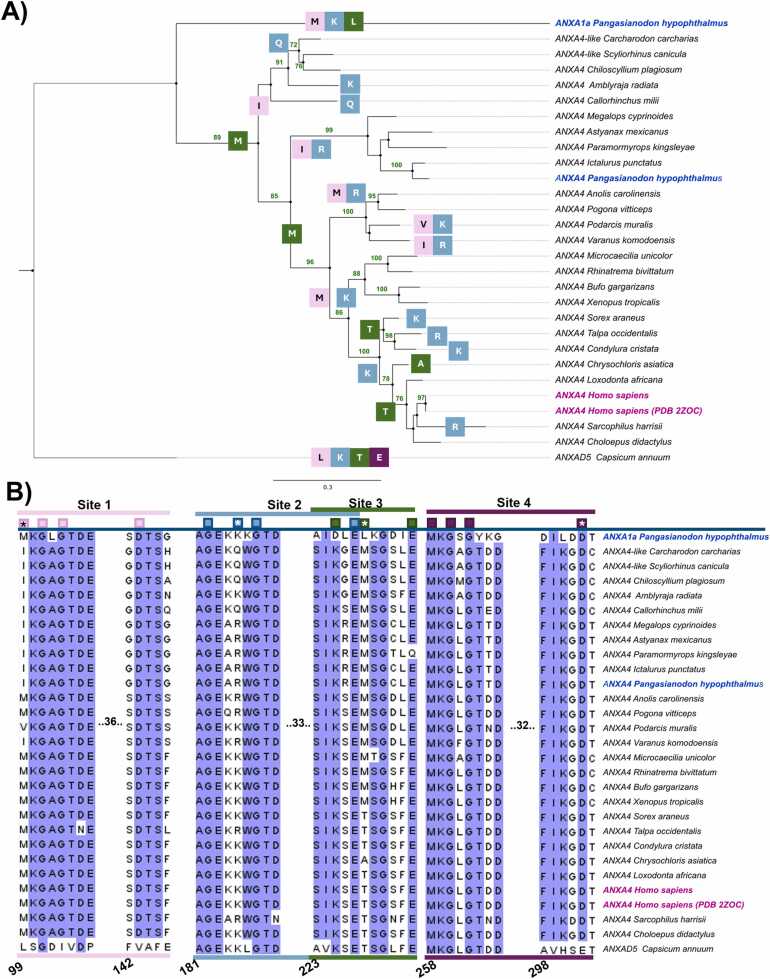
**Sequence conservation of PH-ANXs compared to different vertebrate species.** A) Maximum likelihood tree of 29 ANX sequences, using 1000 bootstraps for support values (shown in green) on IQtree. *Capsicum annum* was used as an outgroup. Changes in Ca^2+^ coordination residues are depicted in boxes at each branch, where pink, blue, green, and purple boxes correspond to sites 1, 2, 3, and 4, respectively. B) MSA of ANX Ca^2+^ site sequences. Squares indicate a Ca^2+^ coordination residue, whereas those with a * indicate variable sites depicted on the phylogenetic tree. Residue numbering is according to ANXA4 (2ZOC).

PH-ANXA1a and PH-ANXA4 were then compared phylogenetically with other vertebrates, including H-ANXA4, to understand the primary sequence variations within canonical and noncanonical Ca^2+^ sites from *P. hypophthalmus* candidates **(**Fig. 4**)**. Phylogenetic analysis revealed that H-ANXA4 evolved from the divergence of aquatic and amphibian clades, making it related yet distant from PH-ANXA1a and PH-ANXA4 sequences (Fig. 4**A**). Furthermore, changes at noncanonical Ca^2+^ coordinating amino acids (NCaa) can be observed based on speciation events (Fig. 4**B**). Within site 1, it can be observed that M99 in H-ANXA4 is maintained for PH-ANXA1a, while the M99I substitution is a transition that occurred after clade separation of fish and amphibian at PH-ANXA4. In the case of H-ANXA4, M acts as a carbonyl anchoring site. M and I are both hydrophobic amino acids, with I being shorter for one carbon, making the I site less rigid. Fish cell membranes are more flexible despite having similar physicochemical characteristics as mammalian cells [78]. This may allow a more flexible Ca^2+^ site, which may explain the M to I substitution.

Within site 2, it can be observed that carbonyl donating K185 in H-ANXA4 is maintained in PH-ANXA1a (Fig. 4**B**). Meanwhile, K185 changes to R within the PH-ANXA4 clade (Fig. 4**A**). These amino acids have been reported to allow a backbone carbonyl Ca^2+^ interaction within EF-hand -Y position, where K and R amino acids appear typical, as well as T, F, Q, Y, and E [79]. Sites 3 and 4 are highly conserved among species, where PH-ANXA1a is the only one that differs at NCaa, indicating that these sites are essential for ANX function along species.

With this analysis, it can be observed that functional motifs are well conserved for PH-ANXs despite primary sequence variation, indicating a convergence phenomenon for Ca^2+^ binding sites [80]. This suggests that PH-ANXA4 and PH-ANXA1a may be adequate candidates to be tested as wound healing agents.

**Fig. 5:**
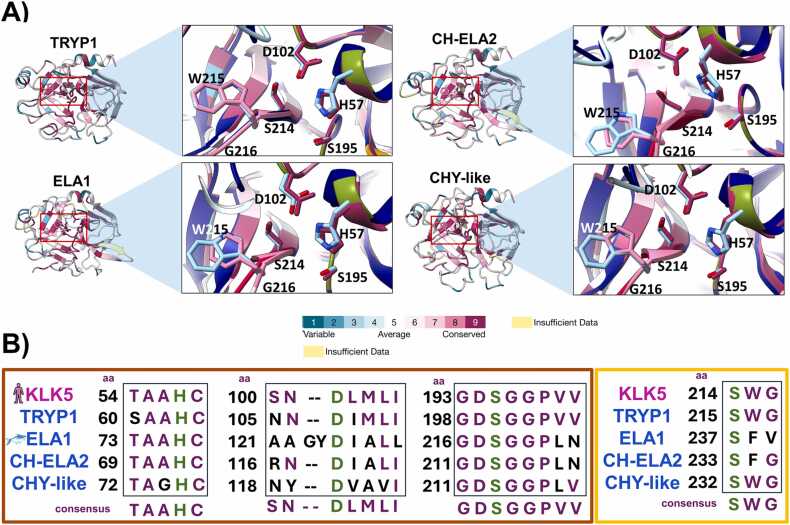
**Structural conservation of PH-SPs.** A) ConSurf analysis and superimposition of PH*-*SP models (ConSurf color scheme) to *H. sapiens* KLK5 (2PSX) as a template (navy blue). Residues corresponding to the catalytic triad (HDS) and substrate binding pocket (SWG) are indicated according to KLK5 numbering [84]. B) Motif conservation of the catalytic triad (orange box) and substrate pocket (yellow box) in human KLK5 and PH*-*SPs. Residues involved in catalysis and substrate binding are shown in green.

As for PH-SPs, conservation was evaluated at the catalytic triad, substrate binding pocket (SPk), and specificity P1 position level, as these determine SP function [81], [82]. ConSurf analysis showed that PH-TRYP1, PH-ELA1, PH-CH-ELA2, and PH-CHY-like maintain structural conservation at disulfide bridges, catalytic triad, and SPk sites (Fig. 5**A**). When structurally compared to H-KLK5, it can be observed that spatial placing for catalytic triad amino acids (H57, D102, and S195) and SPk amino acids is well conserved in all compared PH-SPs (Fig. 5**A**). At the sequence level, the canonical motifs of the H-KLK catalytic triad TAA**H**C and GD**S**GGP [83] are well preserved in all PH-SPs, with slight variations for PH-ELA1. In addition, **D**LML [83] motif variations in PH-SPs (Fig. 5**B**) did not generate a change of D residue position in most of the compared models (Fig. 5**A**).

Only slight spatial variations were observed at PH-ELA D102 and S195. This change may be due to the insertion of a GY amino acid before D102 (Fig. 5**B**). With these results, it can be observed that PH-TRYP1, PH-CH-ELA2, and PH-CHY-like have a similarity with H-KLK5 at the catalytic triad architecture.

Zooming into primary sequence comparison at SPk amino acids (S214, W214, and G216), S214 is maintained in all sequences (Fig. 5**B**), as it is vital to promote D102 polar state during catalysis [81]. Likewise, the binding pocket architecture is maintained despite W214F substitution at PH-ELA1 and PH-CH-ELA2 (Fig. 5**B**), as the substitution is for another hydrophobic amino acid. PH-TRYP1 and HP-CHY-like substrate binding pockets are identical to H-KLK5.

**Fig. 6:**
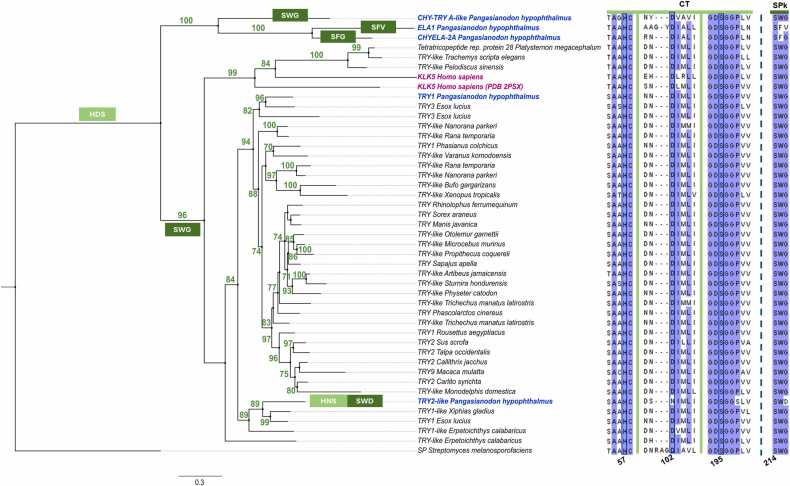
**Sequence conservation of PH-SPs compared to SPs from different vertebrate species.** The tree was constructed using maximum likelihood, using 45 different SP sequences and 1000 bootstraps for support values (shown in green) on IQtree. *Streptomyces melanosporofaciens* was used as an outgroup. Changes in catalytic triad (HDS) and substrate pocket (SWD) residues are represented on light and dark green boxes, respectively. MSA numbering is according to KLK5 (2PSX). CT. catalytic triad; SPk, substrate pocket.

Moreover, it can be observed that the catalytic triad and SPk residues are well conserved among vertebrates (Fig. 6). Such conservation agrees with the described positive selection of active site residues of SP at the genetic level. For instance, AGY and TCN codons, which correspond to the catalytic site serine, are preserved within different SPs [81]. This indicates that SPs are highly significant in vertebrate biology to be well preserved through several taxa, as these proteases are involved in important biological processes, including cell proliferation, migration, and angiogenesis, which are key factors for wound healing [85]. Only a few variations of SPk amino acids could be observed within PH-ELA1 and PH-CH-ELA2 (Fig. 6).

**Fig. 7:**
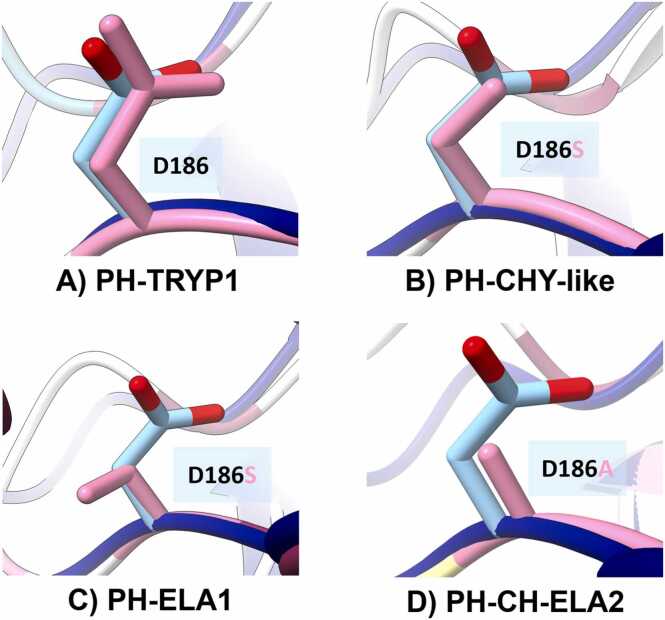
**Variations on position 186 of PH-SP candidates compared with H-KLK 5.** A) HP-TRYP1 D186 leading to K and R interaction preference; B) PH-CHY-like and C) PH-ELA1 D186S leading to F, Y, and W interaction preference; D) PH-CH-ELA2 D186A leading to A, V, and L interaction preference.

Structural analysis of specificity S1 position showed the variations at the residue in position 186 of H-KLK5 (Fig. 7) as the main differences between H-KLK5 and PH-SPs. This position has been reported to provide amino acid specificity to KLK and SP proteins [86], [87], where D, S, and A lead to trypsin, chymotrypsin, and elastase substrate preference, respectively [86], [88]. H-KLK5 and HP-TRYP1 have a D186, making both a trypsin-type SP with a preference cut between positive K and R [89]. This configuration allows H-KLK5 to cleave various substrates involved in different processes of wound healing, such as in the coagulation cascade (fibrinogen and plasminogen), extracellular matrix remodeling (collagen I, II, IV, laminin, and fibronectin), protease activation (MMP9, MMP12, MMP13, and MMP14), and growth factor activation (latent tissue growth factor β 1) [5], [86]. Thus, HP-TRYP1 may be tested *in vitro* to identify its participation during such processes.

Conversely, PH-CHY-like (Fig. 7**B**) and PH-ELA1 (Fig. 7**C**) have a D186S substitution, corresponding to chymotrypsin type SP, with F, Y, and W aromatic amino acid preference [86], [89], [90]. This substrate affinity may be inconvenient for tissue remodeling, as collagens have a very low aromatic amino acid content [91]. Moreover, as this residue enhances collagen self-association ^78^, its catalysis with chymotrypsin-type proteases could only be possible in thermal damage wounds due to collagen denaturation [92]. On the other hand, PH-CHY-like and PH-ELA1 also structurally matched with KLK7, which is also a chymotrypsin-type protease [93] that has been reported to participate in E-cadherin catalysis, relating to keratinocyte migration in wound healing [62]. Therefore, these SP candidates are attractive alternatives to be tested in H-KLK7-related activities.

PH-CH-ELA2′s D186A (Fig. 7**D**) substitution leads to an elastase preference for aliphatic amino acid chains, including hydrophobic amino acids A, V, and L [87], [88], [94]. In wound healing, neutrophil-sourced human elastases act as antibacterial agents. Elastases also catalyze extracellular matrix components, such as elastin, fibronectin, laminin, and collagen IV, indicating their participation in basement membrane formation and reepithelialization processes [95]. Hence, from the PH-SPs, PH-TRYP1 would be the most similar candidate to H-KLK5, with the potential to be evaluated as an adjuvant within different wound healing steps.

Today, debridement formulations mainly focus on the catalytic potential of vegetal and bacterial proteases to eliminate eschar tissue, a mechanism that has been exploited for the last 30 years. In addition, most discovery pipelines rely on labor-intensive bio-guided identification. Homology-based identification can help optimize this by offering adequate selection criteria to identify molecules that will have the functional motifs of those found in human wound healing.

This information can be used to test and develop more selective debridement treatments, as rational selection indicates that topical formulations could include not only PH-SPs as debriding agents but also PH-ANXA4 intervention in inflammation modulation, given that PH-ANXs resemble H-ANXA4, which have been reported to play a role in cell membrane healing (H-ANXA4) and inflammation modulation (H-ANXA1) [66]. To promote potential candidates from *in silico* to functional bioactives in topical formulations, they must be tested at the *in vitro* level in terms of stability, bioactivity, topical delivery, application order of bioactives, and efficiency compared with the already existing formulations.

This study also opens the possibility of identifying biological agents from other organisms that are also fast healers like *P. hypophthalmus* [25] and encountering molecules that may be more resistant to environmental changes, such as basic pH (6.5–8.5) found in chronic wounds [96]. For example, PH-SPs have a calculated pI of 6.86–8.6 (Table S.4), making them suitable for acting in such environments. On the other hand, among PH-ANXs, only PH-ANXA1a has a pI of 7.4, while PH-ANXA4 has a more acidic pI of 5.35, indicating that this agent will require a buffering environment to maintain proper activity.

## 4 Conclusion

Burns and chronic wounds are injuries that represent a physical and economic burden to patients. Due to their prolonged healing time, it is pertinent to find new, cost-effective solutions. The present study identified six potential wound healing agents, which were genetically validated within the skin of *P. hypophthalmus*. In addition, functional motif sequence and structural analysis revealed that PH-ANX binding sites are well conserved between vertebrates, making GTxGD sites a marker for targeting ANX candidates with possible PS binding capacity. Further studies are needed to understand the effect of NCaa at Ca^2+^ sites and the EF-hands-like binding site. Additionally, SP motif analysis showed that PH-SP catalytic triad structure and motifs are well preserved compared to H-KLK5. Nonetheless, the amino acid at position 186 will determine substrate specificity, with PH-TRYP1 as the SP that shares the highest structural similarity to H-KLK5. These discoveries provide additional information to the development of rational topical formulations for debridement and wound healing in the future. For instance, there is a guided context on which molecular actions of H-ANXs and H-SPs should be tested in *in vitro* models for PH-SPs and PH-ANXs due to their structural homology and functional motif homology.

## Declaration of Competing Interest

The authors declare that they have no known competing financial interests or personal relationships that could have appeared to influence the work reported in this paper.

## Footnotes

Appendix A Supplementary data associated with this article can be found in the online version at doi:10.1016/j.csbj.2024.10.015.
