# Supplementary material for: Homology-based identification and structural analysis of Pangasius hypophthalmus Annexins and Serine proteases to search molecules for wound healing applications
Source: Comput Struct Biotechnol J. 2024 Oct 11;23:3680–91. doi: 10.1016/j.csbj.2024.10.015 (PMC11539086; doi:10.1016/j.csbj.2024.10.015)
Supplement: Supplementary file 1 — Supplementary material [file mmc1.docx]

1. **Appendix A. Supplementary Information**

**Table S.1 UniProtKB dataset of annotated extracellular ANX from teleost species used for TBLASN. Domain annotation was held through Prosite. AA, amino acid**

| **Protein ID** | **Specie** | **Protein name** | **AA**  **Domain range** | | **Domain name** | **Domain quantity** |
| --- | --- | --- | --- | --- | --- | --- |
| Q804H2 | *Danio rerio* | Annexin 1a | 37 | 108 | ANNEXIN_2 | 4 |
|  |  |  | 109 | 180 |  |  |
|  |  |  | 192 | 264 |  |  |
|  |  |  | 268 | 339 |  |  |
| Q7T3A8 |  | Annexin 1a | 37 | 108 |  | 4 |
|  |  |  | 109 | 180 |  |  |
|  |  |  | 192 | 264 |  |  |
|  |  |  | 268 | 339 |  |  |
| Q804H1 |  | Annexin 1b | 38 | 109 |  | 4 |
|  |  |  | 110 | 181 |  |  |
|  |  |  | 193 | 265 |  |  |
|  |  |  | 269 | 340 |  |  |
| B8JLZ3 |  | Annexin 1b | 38 | 109 |  | 4 |
|  |  |  | 110 | 181 |  |  |
|  |  |  | 193 | 265 |  |  |
|  |  |  | 269 | 340 |  |  |
| A0A0R4ICD6 |  | Annexin 1c | 37 | 85 |  | 3 |
|  |  |  | 97 | 169 |  |  |
|  |  |  | 173 | 244 |  |  |
| Q32PQ2 |  | Annexin 1c | 37 | 108 |  | 3 |
|  |  |  | 135 | 207 |  |  |
|  |  |  | 211 | 282 |  |  |
| Q804H0 |  | Annexin 1c | 37 | 108 |  | 4 |
|  |  |  | 109 | 180 |  |  |
|  |  |  | 192 | 264 |  |  |
|  |  |  | 268 | 339 |  |  |
| A0A2R8RXX8 |  | Annexin 1d | 37 | 85 |  | 3 |
|  |  |  | 97 | 169 |  |  |
|  |  |  | 173 | 244 |  |  |
| A0A0G2L5W0 |  | Annexin 1d | 37 | 107 |  | 4 |
|  |  |  | 108 | 179 |  |  |
|  |  |  | 191 | 263 |  |  |
|  |  |  | 267 | 338 |  |  |
| Q568I4 |  | Annexin 1d | 36 | 107 |  |  |
|  |  |  | 108 | 179 |  |  |
|  |  |  | 191 | 263 |  |  |
|  |  |  | 267 | 338 |  |  |
| O93446 | *Oryzias latipes* | *Annexin 1* | 34 | 105 | ANNEXIN_2 | 4 |
|  |  |  | 106 | 177 |  |  |
|  |  |  | 187 | 259 |  |  |
|  |  |  | 263 | 334 |  |  |

**Table S.2 Uniport dataset of annotated extracellular SP from teleost species used for TBLASN analysis. Domain annotation was held through Prosite. AA, amino acid; IGFBP_N_2, insulin-like growth factor-binding protein (IGFBP) N-terminal domain profile; KAZAL_2,** **Kazal domain profile; PDZ**, **PDZ domain profile**

| **Protein ID** | **Specie** | **Protein name** | **AA**  **Domain range** | | **Domain name** | **Total units** |  |
| --- | --- | --- | --- | --- | --- | --- | --- |
| Q6GMI0 | *Danio rerio* | Serine protease HTRA1A | 203 | 363 | peptidase S1C family/degP_htrA_DO super family | 3 |  |
|  |  |  |  |  |  |  |  |
|  |  |  | 27 | 111 | IGFBP_N_2; |  |  |
|  |  |  | 102 | 155 | KAZAL_2 |  |  |
|  |  |  | 410 | 465 | PDZ |  |  |
| A9JRB3 |  | Serine protease HTRA1B | 200 | 360 | peptidase S1C family/degP_htrA_DO super family | 3 |  |
|  |  |  |  |  |  |  |  |
|  |  |  | 25 | 109 | IGFBP_N_2; |  |  |
|  |  |  | 100 | 153 | KAZAL_2 |  |  |
|  |  |  | 407 | 473 | PDZ |  |  |
| Q7SIG3 | *Salmo salar* | Elastase-1 | 1 | 236 | TRYPSIN_DOM/Peptidase 1 | 1 |  |
| P35031 |  | Trypsin-1 | 21 | 240 |  |  |  |
| P35032 |  | Trypsin-2 | 10 | 229 |  |  |  |
| P35033 |  | Trypsin-3 | 16 | 236 |  |  |  |
| P47796 | *Gadus morhua* | Chymotrypsin A | 34 | 261 |  |  |  |
| P16049 |  | Trypsin-1 | 20 | 239 |  |  |  |
| Q91041 |  | Trypsin-10 | 20 | 239 |  |  |  |
| P80646 |  | Chymotrypsin B | 16 | 243 |  |  |  |
| P35034 | *Pleuronectes platessa* | Trypsin | 23 | 247 |  |  |  |

**Table S.3 DALI results for A) ANX and B) SP candidates. LALI, length of aligned residues; NRES, total number of residues; Z, Z-score; ID, identity**

**A)**

| **Query** | **Hit chain** | **Z** | **RMSD** | **LALI** | **NRES** | **ID%** | **PDB description** |
| --- | --- | --- | --- | --- | --- | --- | --- |
| Annexin A4 | 1axn-A | 45.6 | 1.3 | 321 | 323 | 50 | ANXA3 |
|  | 2zoc-A | 45.6 | 0.9 | 317 | 319 | 60 | ANXA4 |
|  | 1w45-A | 41.1 | 1.4 | 312 | 320 | 48 | ANXA8 |
| Annexin A1a | 2zoc-A | 44.4 | 1.1 | 318 | 319 | 47 | ANXA4 |
|  | 1anw-A | 39.5 | 2.1 | 316 | 319 | 45 | ANXA5 |

**B)**

| **Query** | **Hit chain** | **Z** | **RMSD** | **LALI** | **NRES** | **ID%** | **PDB description** |
| --- | --- | --- | --- | --- | --- | --- | --- |
| Trypsin 1 | 2psx-A | 37.1 | 1 | 222 | 227 | 47 | KLK5 |
|  | 5ms3-A | 36.9 | 1.2 | 222 | 228 | 44 | KLK8 |
|  | 2qxg-A | 36.1 | 1.1 | 221 | 225 | 45 | KLK7 |
|  | 6nvb-B | 35.6 | 1.4 | 221 | 224 | 39 | KLK4 |
| Elastase-1-like | 5ms3-A | 34.6 | 1.3 | 222 | 228 | 35 | KLK8 |
|  | 2psx-A | 33.4 | 1.2 | 221 | 227 | 25 | KLK5 |
|  | 2qxga-A | 32.3 | 1.4 | 218 | 225 | 31 | KLK7 |
|  | 6nvb-B | 32.3 | 1.7 | 219 | 224 | 31 | KLK4 |
|  | 5lpe-A | 24.7 | 2.1 | 199 | 204 | 25 | KLK10 |
|  | 1gvl-A | 24.4 | 2.4 | 203 | 223 | 29 | KLK6 |
| Chymotrypsin A-like | 5ms3-A | 32.6 | 1.5 | 220 | 228 | 34 | KLK8 |
|  | 2psx-A | 32.2 | 1.4 | 221 | 227 | 31 | KLK5 |
|  | 6nvb-B | 31.1 | 1.7 | 218 | 224 | 28 | KLK4 |
|  | 2qxg-A | 31.1 | 1.6 | 217 | 225 | 32 | KLK7 |
|  | 5lpe-A | 26.6 | 2 | 200 | 204 | 34 | KLK10 |
|  | 1gvl-A | 25.4 | 2.6 | 206 | 223 | 31 | KLK6 |
| Chymotrypsin-like elastase isoform X2 | 2psx-A | 34.4 | 1.2 | 223 | 227 | 32 | KLK5 |
|  | 5ms3-A | 34 | 1.3 | 221 | 228 | 34 | KLK8 |
|  | 6nvb-B | 33.2 | 1.5 | 221 | 224 | 38 | KLK4 |
|  | 2qxg-A | 32.4 | 1.4 | 219 | 225 | 34 | KLK7 |

**Table S.4 Theoretical calculations of molecular weight (MW) and isoelectric point of *P. hypophthalmus* SP and ANX computed through Expasy Compute pI/Mw**

| **Candidate protein** | **Protein accession** | **Calculated MW (kDa)** | **Calculated isoelectric point** |
| --- | --- | --- | --- |
| Trypsin 1 | XP_026769278.1 | 26.48 | 6.86 |
| Elastase-1-like | XP_026788217.1 | 29.29 | 8.4 |
| Chymotrypsin-like elastase family member 2A isoform X2 | XP_026776291.1 | 28.79 | 8.66 |
| Chymotrypsin A-like | XP_026774546.1 | 27.78 | 8.5 |
| Annexin A1a | XP_026795943.1 | 37.289 | 7.48 |
| Annexin A4 | XP_026798592.1 | 35.81 | 5.35 |

**Figure S.1 PH-trypsin 1 AlphaFold model quality parameters. A) MSA depth, B) pLDDT, and C) predicted alignment error, where blue and red represent low and high error rate, respectively**

**
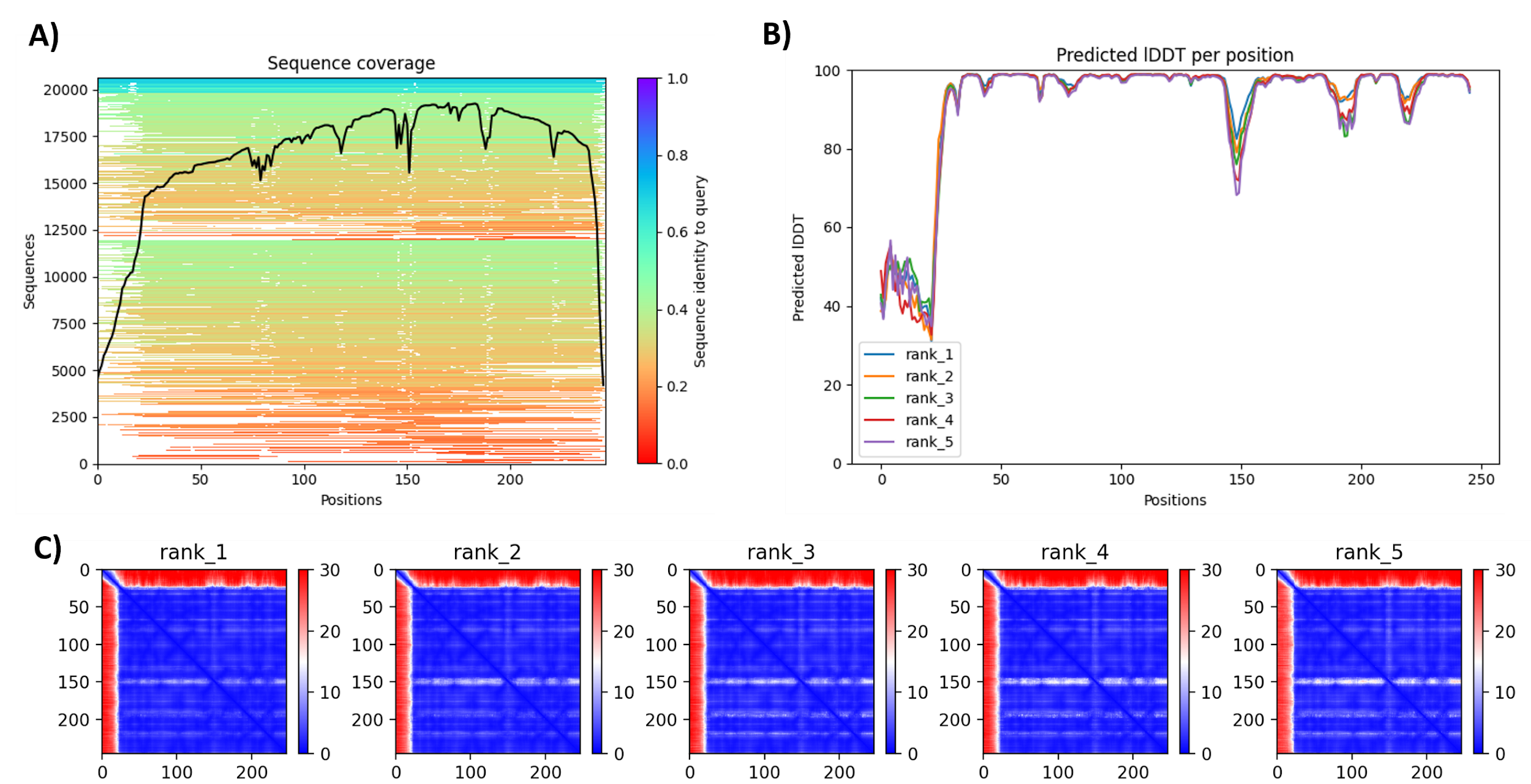
**

**Figure S.2 PH-elastase-1 AlphaFold model quality parameters. A) MSA depth, B) pLDDT, and C) predicted alignment error, where blue and red represent low and high error rate, respectively**

**
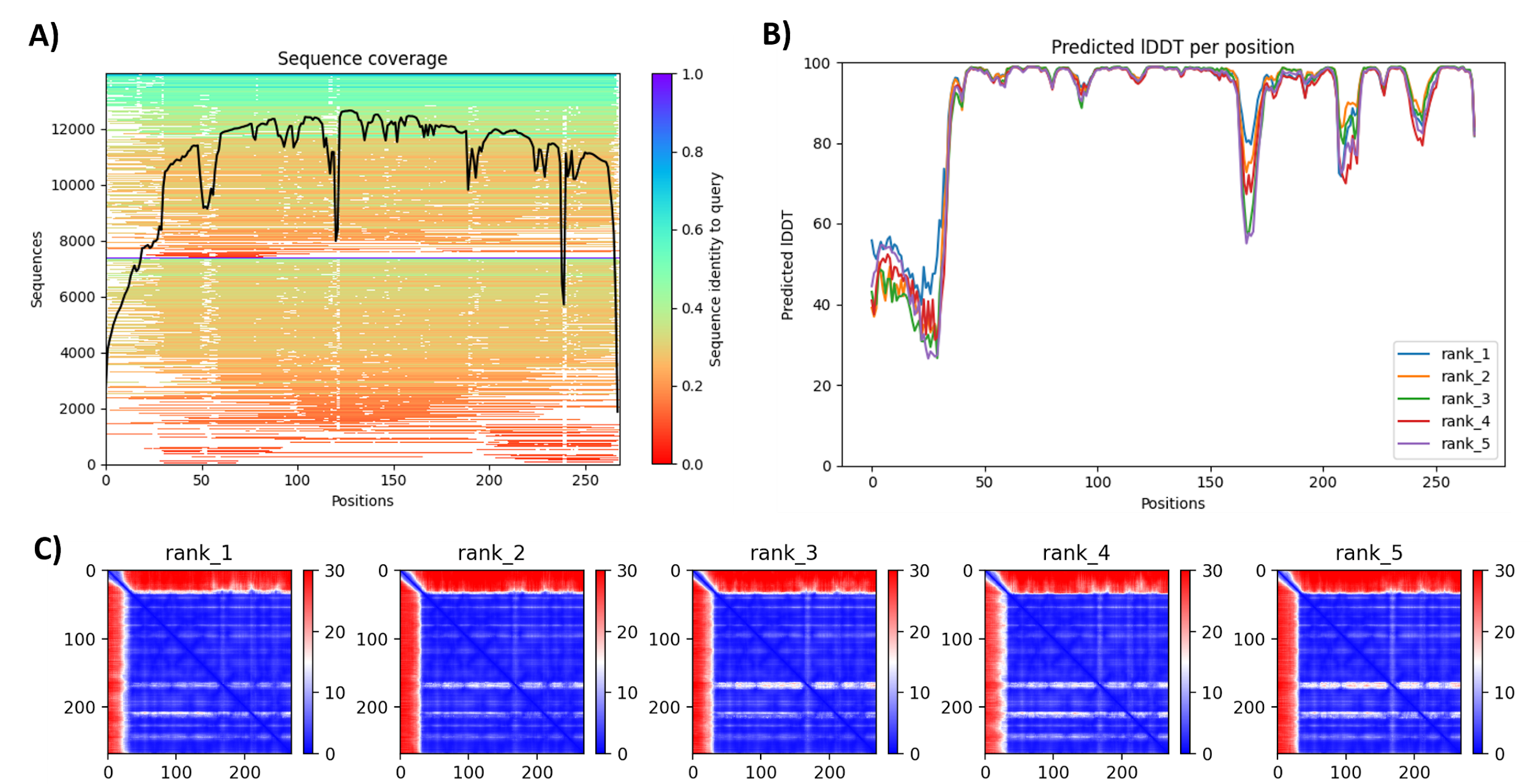
**

**Figure S.3 PH-chymotrypsin-like elastase family member 2A isoform X2 AlphaFold model quality parameters. A) MSA depth, B) pLDDT, and C) predicted alignment error, where blue and red represent low and high error rate, respectively**

**
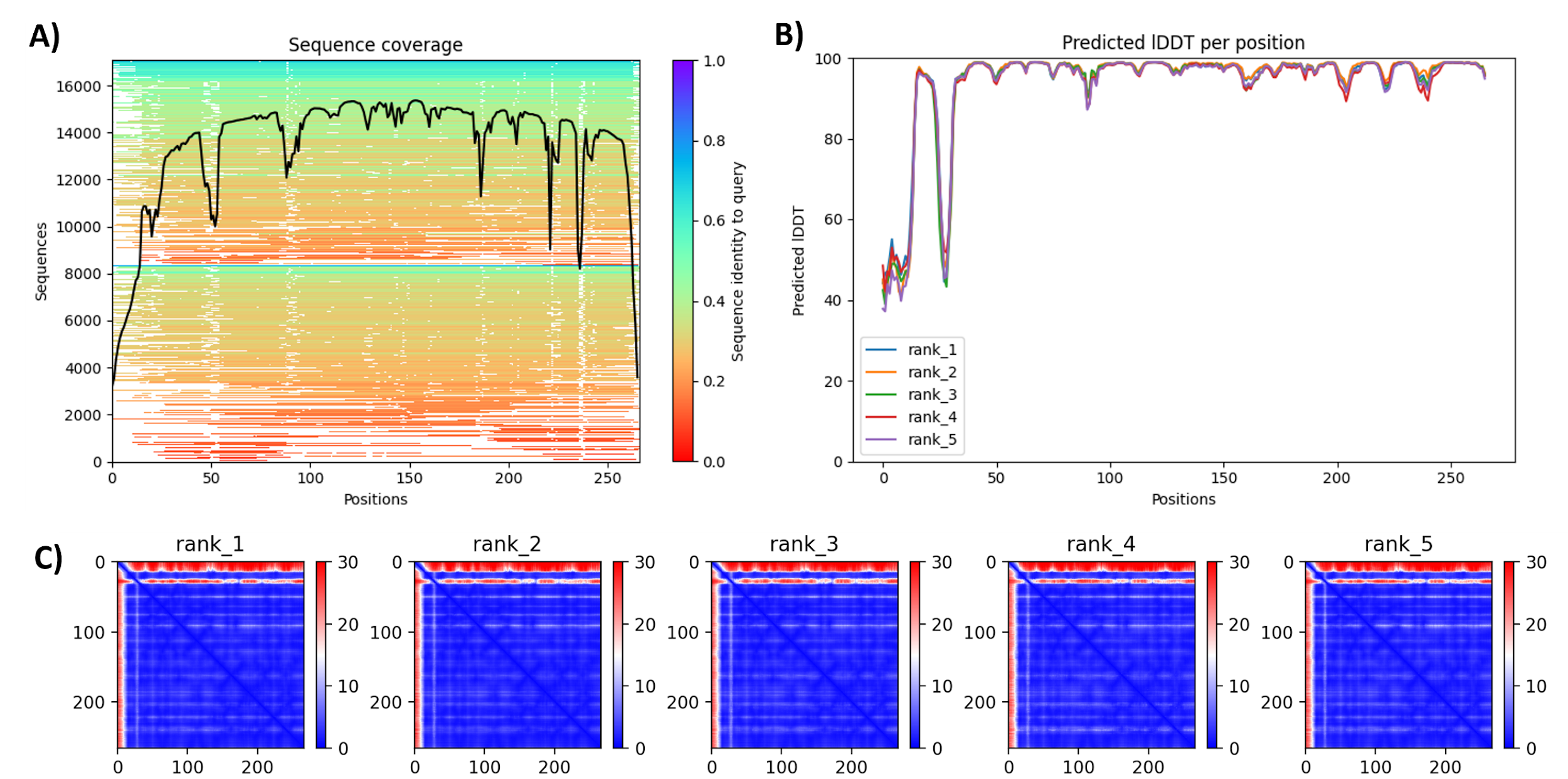
**

**Figure S.4 PH-chymotrypsin-A like AlphaFold model quality parameters. A) MSA depth, B) pLDDT, and C) predicted alignment error, where blue and red represent low and high error rate, respectively**

**
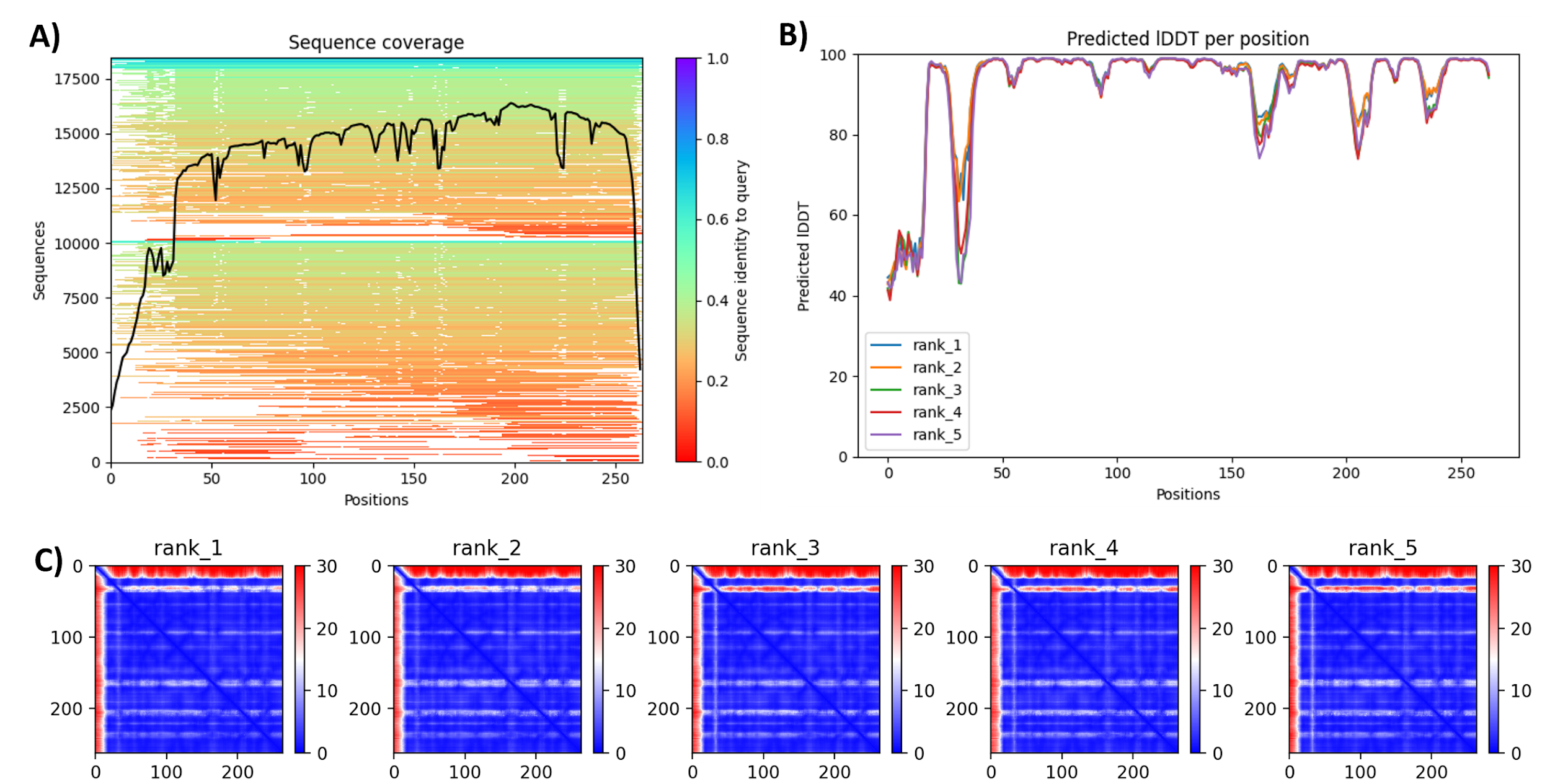
**

**Figure S.5 PH-annexin A4 AlphaFold model quality parameters. A) MSA depth, B) pLDDT, and C) predicted alignment error, where blue and red represent low and high error rate, respectively**

**
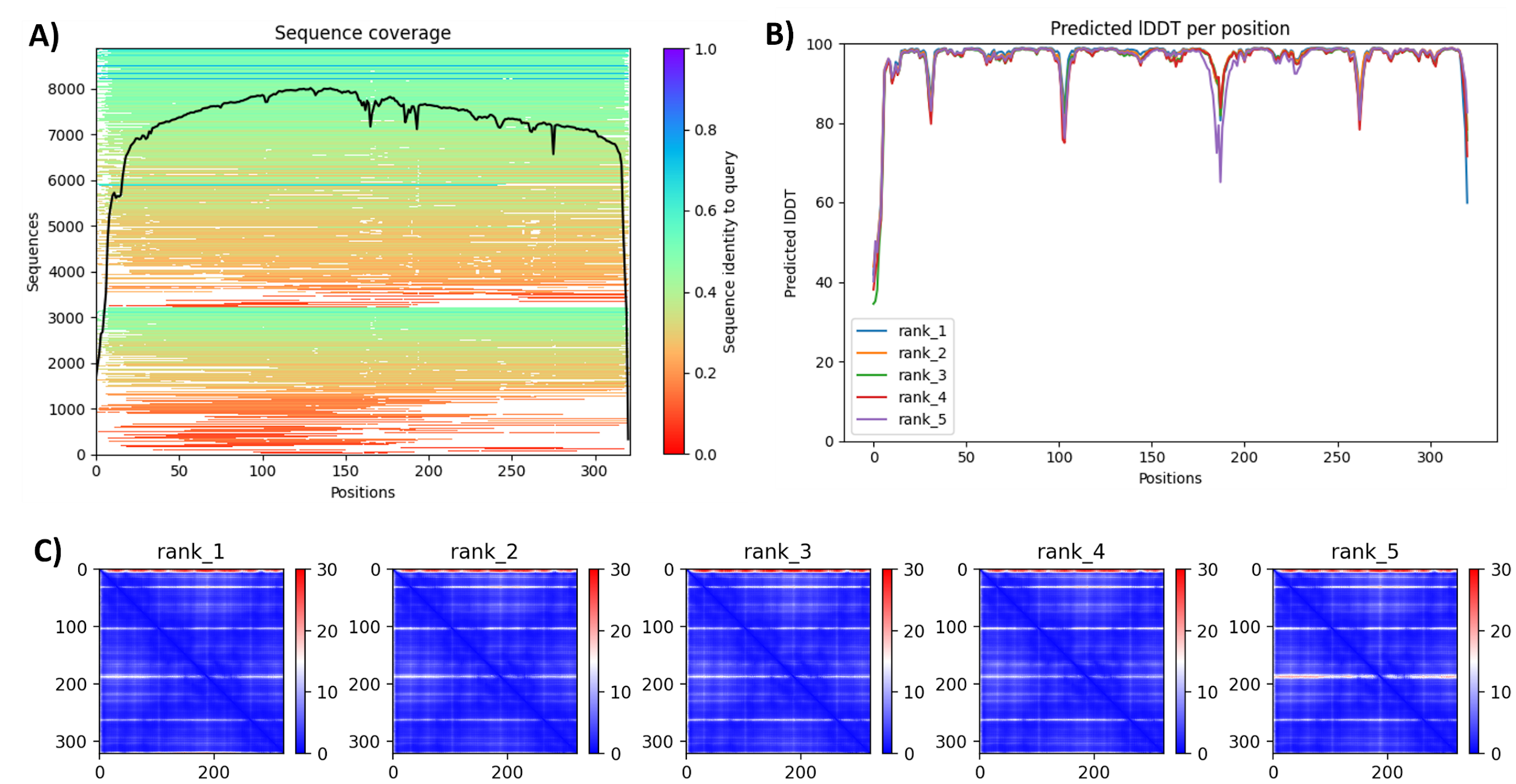
**

**Figure S.6 PH-annexin A1a AlphaFold model quality parameters. A) MSA depth, B) pLDDT, and C) predicted alignment error, where blue and red represent low and high error rate, respectively**

**
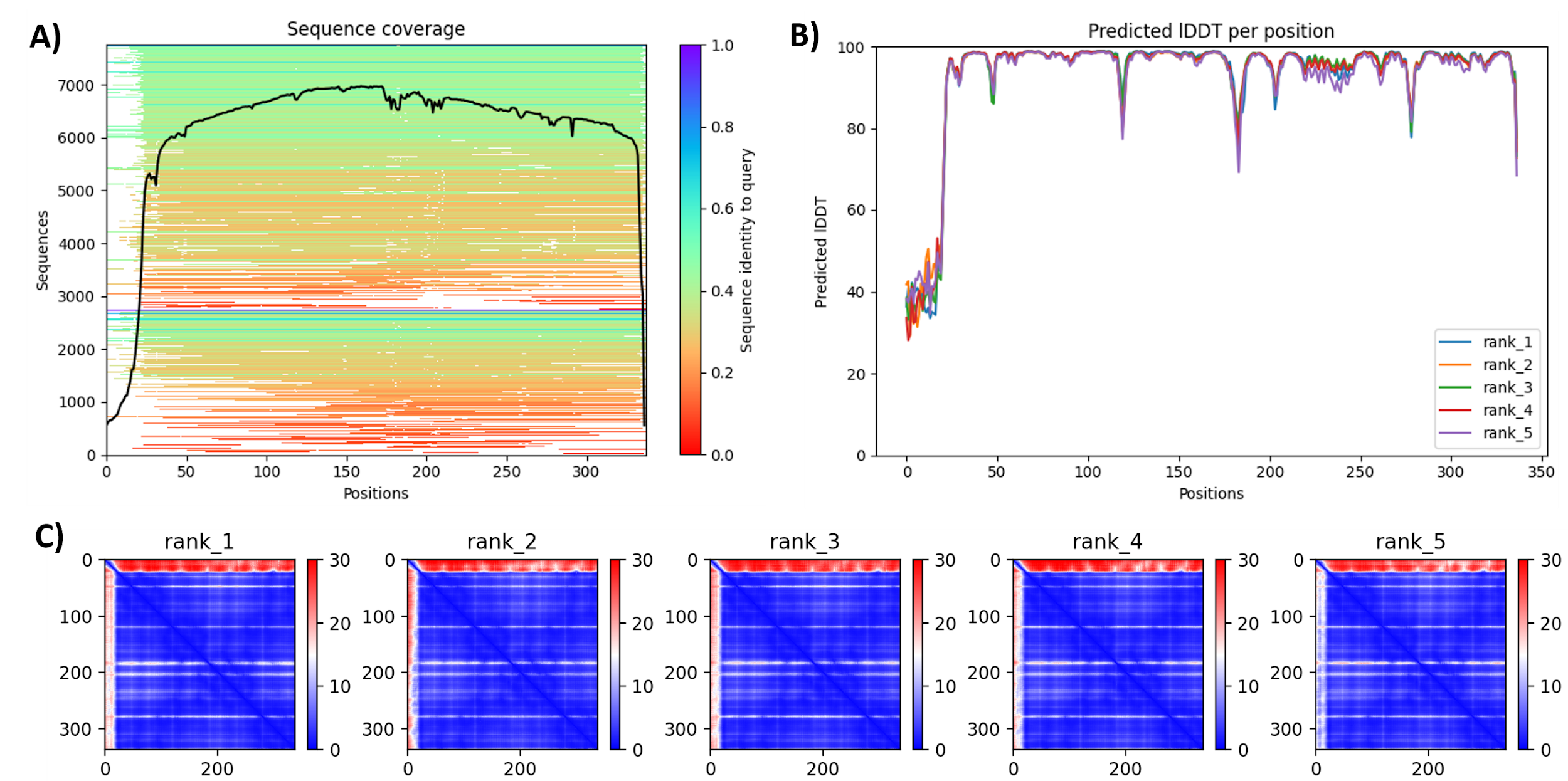
**

**Figure S.7 Vertebrate annexin multiple sequence alignment. Blue color intensity represents sequence conservation up to 90% identity**


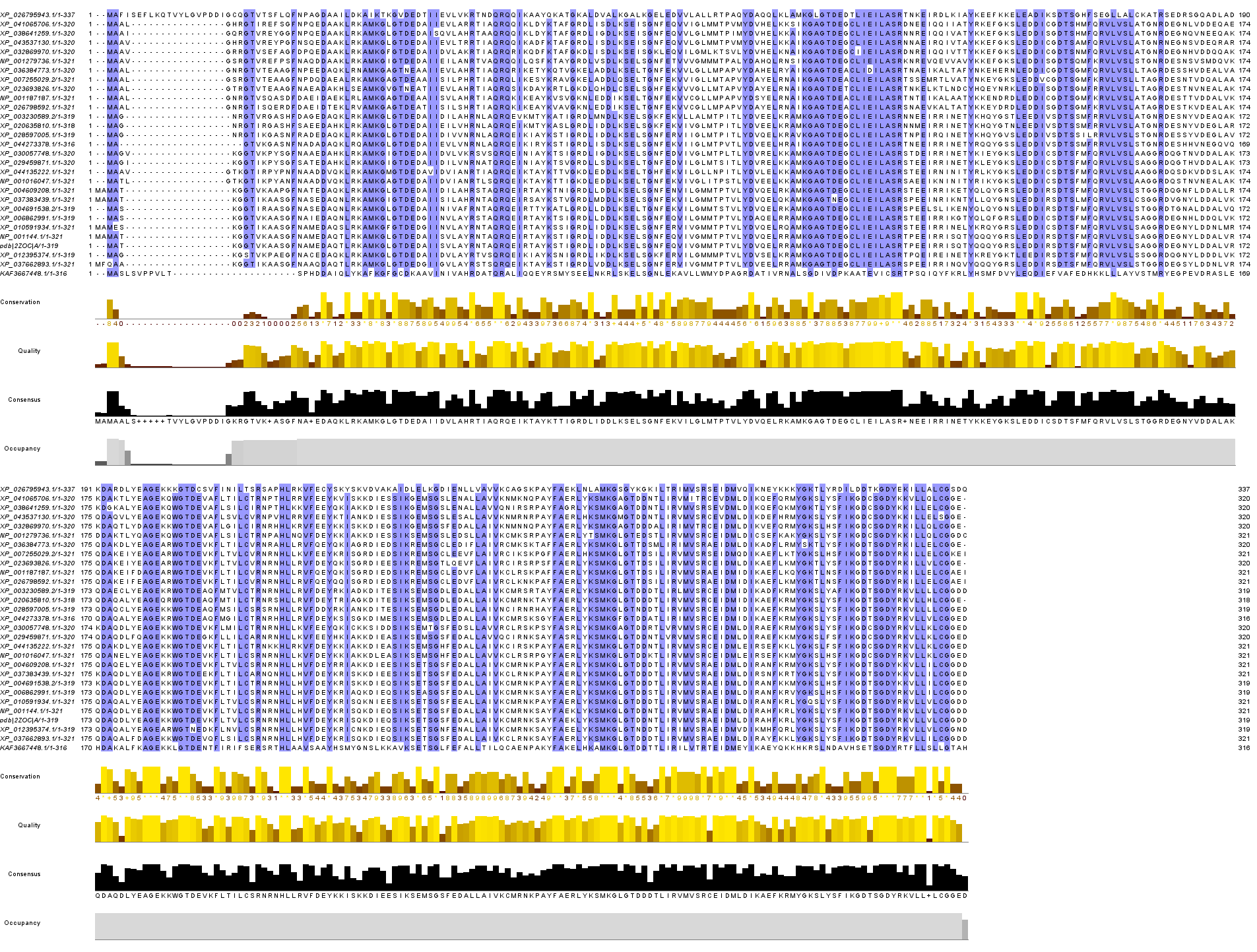


**Figure S.8 Vertebrate serine protease multiple sequence alignment. Blue color intensity represents sequence conservation up to 90% identity**

**
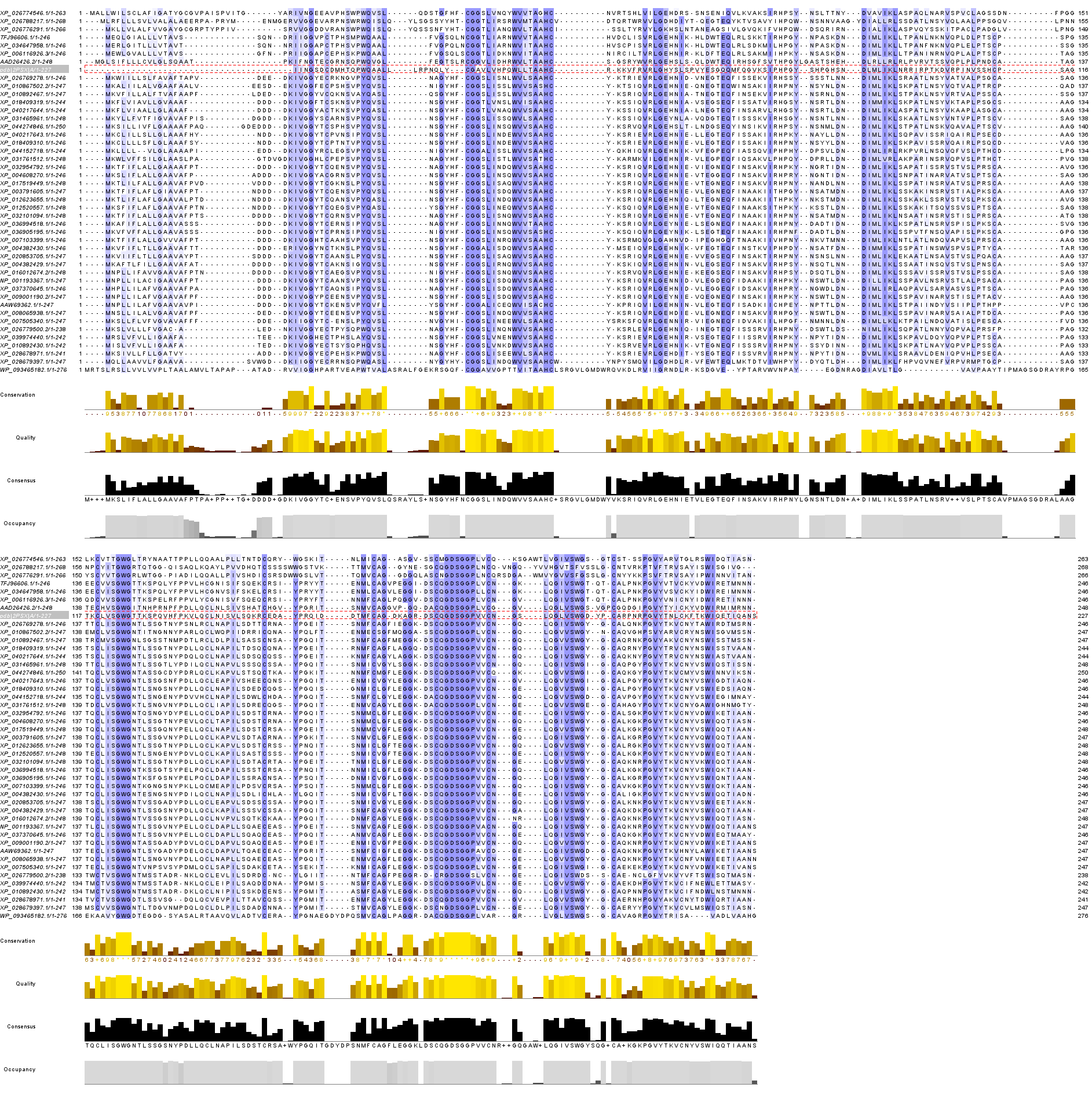
**
